# Supplementary material for: Gut Microbiota-Derived l-Histidine/Imidazole Propionate Axis Fights against the Radiation-Induced Cardiopulmonary Injury
Source: Int J Mol Sci. 2021 Oct 23;22(21):11436. doi: 10.3390/ijms222111436 (PMC8584084; doi:10.3390/ijms222111436)

Supplementary information

1. Materials and methods

1.1 Western blotting

Total protein lysates were obtained from the tissues using RIPA (Solarbio, China) buffer at 4°C for 30 min and centrifuged at 12,000 g and 4°C for 15 min. Tissue lysates were separated by 12% SDS-PAGE, and then transferred onto polyvinylidene fluoride (PVDF) membranes. The membranes were blocked with 5% nonfat milk and incubated with primary antibodies. The list of antibodies and the uncut map of the protein are in Supplementary file.

1.2 Quantitative Real-Time Polymerase Chain Reaction (qRT-PCR)

Cells in each group using Trizol reagent RNA extractant. The harvested cells were treated with chloroform, isopropanol, and 75% ethanol, and RNA was extracted. The RNA concentration of each group was determined, and cDNAs were synthesized using a reverse transcription kit and diluted four times. The diluted cDNA was mixed with primers, SYBR Green, and RNase-free water (dH_2O) in a 20-ll PCR reaction for RT-PCR detection. The primers are listed in Supplementary Table 1.

1.3 Phalloidin staining

In the culture plate, the cover glass with the climbed cells was soaked with PBS for 3 times; Fix the slides with 4% paraformaldehyde for 15 minutes, and wash the slides 3 times with PBS for 3 minutes each time; Drop the diluted phalloidin staining solution (5μg/mL), incubate at 37°C for 1 hour, wash with PBST 3 times, 3 minutes each time; Add DAPI dropwise and incubate in the dark for 5 minutes to stain the specimen, PBST 5min×4 times to wash away the excess DAPI; Use absorbent paper to absorb the liquid on the slide, mount the slide with mounting solution containing anti-fluorescence quencher, and then observe and collect the image under a fluorescence microscope.

1.4 Masson Staining and Sirius Red Staining

The tissue is fixed in 10% formalin fixative, and dehydrated and embedded routinely; Paraffin sections are deparaffinized to water; Wash with tap water and distilled water in turn; Stain the nucleus with Regaud hematoxylin stain or Weigert hematoxylin for 5-10 minutes; Wash with distilled water; Use Masson Ponceau acid complex red solution for 5-10 minutes; Soak for a while with 2% glacial acetic acid aqueous solution; 1% phosphomolybdic acid aqueous solution differentiated for 3-5 min; Without washing, directly dye with aniline blue or light green solution for 5 minutes; Soak for a while with 0.2% glacial acetic acid aqueous solution; 95% alcohol, anhydrous alcohol, transparent xylene, sealing with neutral gum. The Sirius Red Staining was Stain with Sirius Red Staining Solution for 1 hour; Rinse with running water to remove the dye solution on the surface of the slice; Mayer hematoxylin staining solution stains the nucleus for 8-10 minutes.

1.5 lung coefficient

Lung coefficient = wet lung weight / body weight *100%

1.6 Gut-microbiota bacterial diversity analysis and untargeted metabolomics

(1) Bacterial diversity analysis. Bacterial diversity analysis was conducted as

described previously. Stool samples were freshly collected from two independent experiments and stored at -80°C until use. DNA was extracted from the stool using the Power Faecal® DNA Isolation Kit (MoBio Carlsbad, CA USA). Then, mixture PCR products were purified with Qiagen Gel Extraction Kit (Qiagen, Germany). The 16S ribosomal RNA (rRNA) V4 was amplified used specific primer. Sequencing libraries were generated using TruSeq® DNA PCR-Free Sample Preparation Kit (Illumina, USA). The library quality was assessed on the Qubit@ 2.0 Fluorometer (Thermo Scientific) and Agilent Bioanalyzer 2100 system. At last, the library was sequenced on an IlluminaHiSeq2500 platform and 250bp paired-end reads were generated. Sequences analysis was performed by Uparse software (Uparse v7.0.1001, http://drive5.com/uparse/). Sequences with ≥97% similarity were assigned to the same OTUs. Briefly, each cohort contains 12 mice, and 6 mice share one cage. The primers are listed in Supplementary Table 1.

(2) Untargeted metabolomics—metabolite extraction. Feces were individually grounded with liquid nitrogen and the homogenate was suspended with prechilled 80% methanol and 0.1% formic acid by well vortexing. The samples were incubated on ice for 5 min and then were centrifuged at 15000 rpm, 4°C for 5 min. A some of supernatant was diluted to final concentration containing 60% methanol by LC-MS grade water. The samples were subsequently transferred to a fresh Eppendorf tube with 0.22μm filter and then were centrifuged at 15000g, 4°C for 10min. Finally, the filtrate was injected into the LC-MS/MS system analysis.

(3) Untargeted metabolomics—UHPLC-MS/MS analysis. LC-MS/MS analyses were performed using a Vanquish UHPLC system (Thermo Fisher) coupled with an Orbitrap Q Exactive HF-X mass spectrometer (Thermo Fisher). Samples were injected onto an Hyperil Gold column (100 × 2.1mm, 1.9μm) using a 16-min linear gradient at a flow rate of 0.2mL/min. The eluents for the positive polarity mode were eluent A (0.1% FA in Water) and eluent B (Methanol). The eluents for the negative polarity mode were eluent A (5 mM ammonium acetate, pH 9.0) and eluent B (Methanol). The solvent gradient was set as follows: 2% B, 1.5min; 2-100% B, 12.0 min; 100% B, 14.0min; 100-2% B, 14.1min; 2% B, 16min. Q Exactive HF-X mass spectrometer was operated in positive/negative polarity mode with spray voltage of 3.2kV, capillary temperature of 320°C, sheath gas flow rate of 35arb and aux gas flow rate of 10arb.

(4) Untargeted metabolomics—data analysis. The raw data files generated by UHPLC-MS/MS were processed using the Compound Discoverer 3.0 (CD 3.0, Thermo Fisher) to perform peak alignment, peak picking, and quantitation for each metabolite. The main parameters were set as follows: retention time tolerance, 0.2 minutes; actual mass tolerance, 5ppm; signal intensity tolerance, 30%; signal/noise ratio, 3; and minimum intensity, 100000. After that, peak intensities were normalized to the total spectral intensity. The normalized data was used to predict the molecular formula based on additive ions, molecular ion peaks and fragment ions. And then peaks were matched with the mzCloud (https://www.mzcloud.org/) and ChemSpider (http://www.chemspider.com/) database to obtain the accurate qualitative and relative quantitative results.

2. Supplementary table 1

List of primers used in this paper.

Gene	Primer	Sequence (5′-3′)
**Primers for PCR**		
GAPDH	forward	TGTTTCCTCGTCCCGTAGA
	reverse	CAATCTCCACTTTGCCACTG
TNFα	forward	TTCTCATTCCTGCTTGTGGCA
	reverse	ACTTGGTGGTTTGCTACGACG
IL-1	forward	TTGAAGAAGAGCCCATCCTC
	reverse	CAGCTCATATGGGTCCGAC
NF-KB	forward	GAAGCACGAATGACAGAGGC
	reverse	GCTTGGCGGATTAGCTCTTTT
TGF-beta1	forward	GGCCAGATCCTGTCCAAGC
	reverse	GTGGGTTTCCACCATTAGCAC
**Primers for sequencing**		
515F		GTGCCAGCMGCCGCGGTAA
806R		GGACTACHVGGGTWTCTAAT

3. Supplementary figures and figure legends

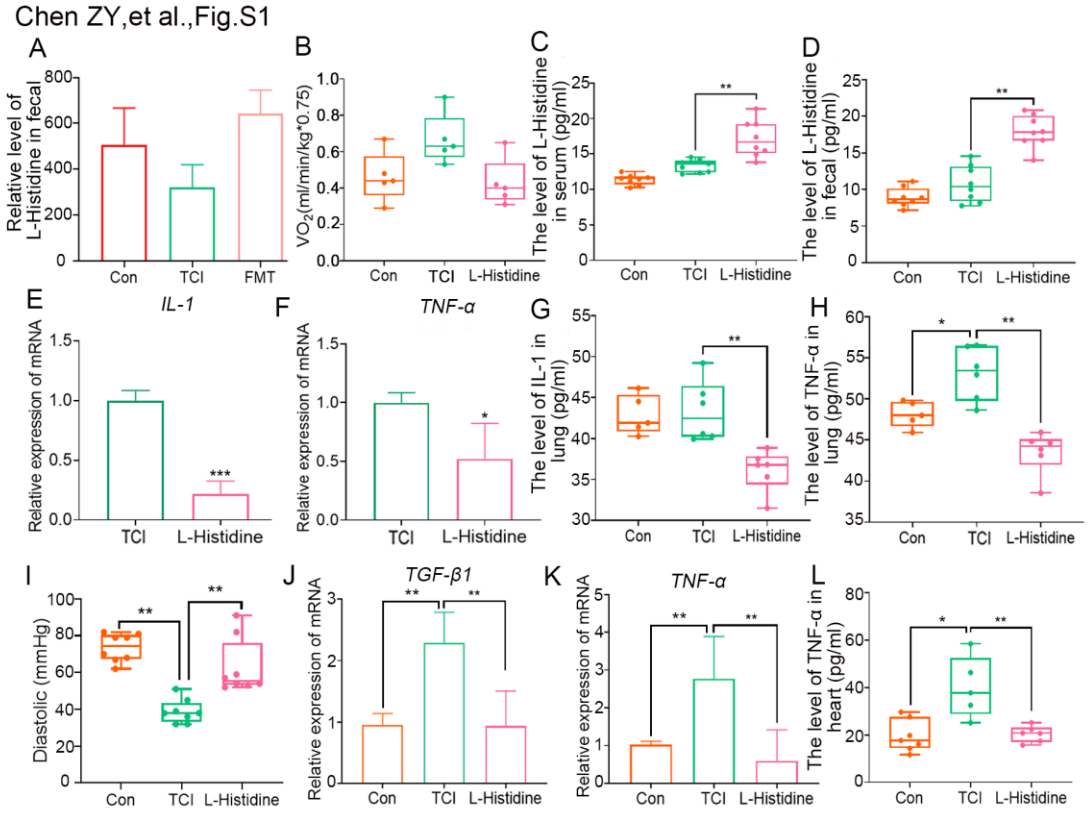

Figure S1. Gut microbiota-derived L-Histidine improves radiation toxicity in lung and heart. (A) The relative of L-Histidine in fecal were assessed by MS. (B) The intake of VO_2 in each group mice in 24 hours (n=6). (C) The L-Histidine expression in serum of each group mice (n=8). (D) The L-histidine expression in fecal of each group mice (n=8). (E, F) The IL-1 and

TNF-α levels in lung tissues of each group mice were assessed by q-PCR. (G, H) The IL-1 and TNF-α levels in lung tissues of each group mice were assessed by ELISA (n=6). (I) The diastolic of each group mice (n=8). (J, K) The TGF-β1 and TNF-α levels in heart tissues of each group mice were assessed by q-PCR. (L) The TNF-α levels in heart tissues of each group mice were assessed by ELISA. ($^{*}p < 0.05$, $^{**}p < 0.01$ and $^{***}p<0.001$; Student's *t*-test)

Chen ZY,et al.,Fig.S2

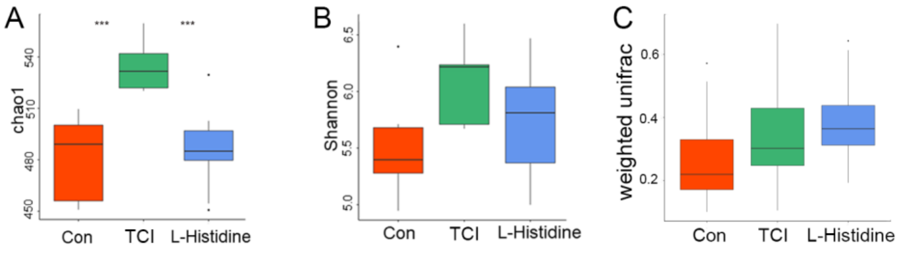

Figure S2. L-Histidine shapes the gut microbiota configuration after chest local irradiation. (A, B) The α-diversity of gut bacteria was measured by 16S rRNA high-throughput sequencing. In detail, the data was represented as the chao1 species number (A) and shannon diversity index (B). (C) The β-diversity of enteric bacteria was compared by weighted_ unifrac analysis. (Statistically significant differences are indicated: Wilcoxon rank sum test, n= 6, $^{*}p < 0.05$, $^{**}p < 0.01$ and $^{***}p < 0.001$).

Chen ZY,et al.,Fig.S3

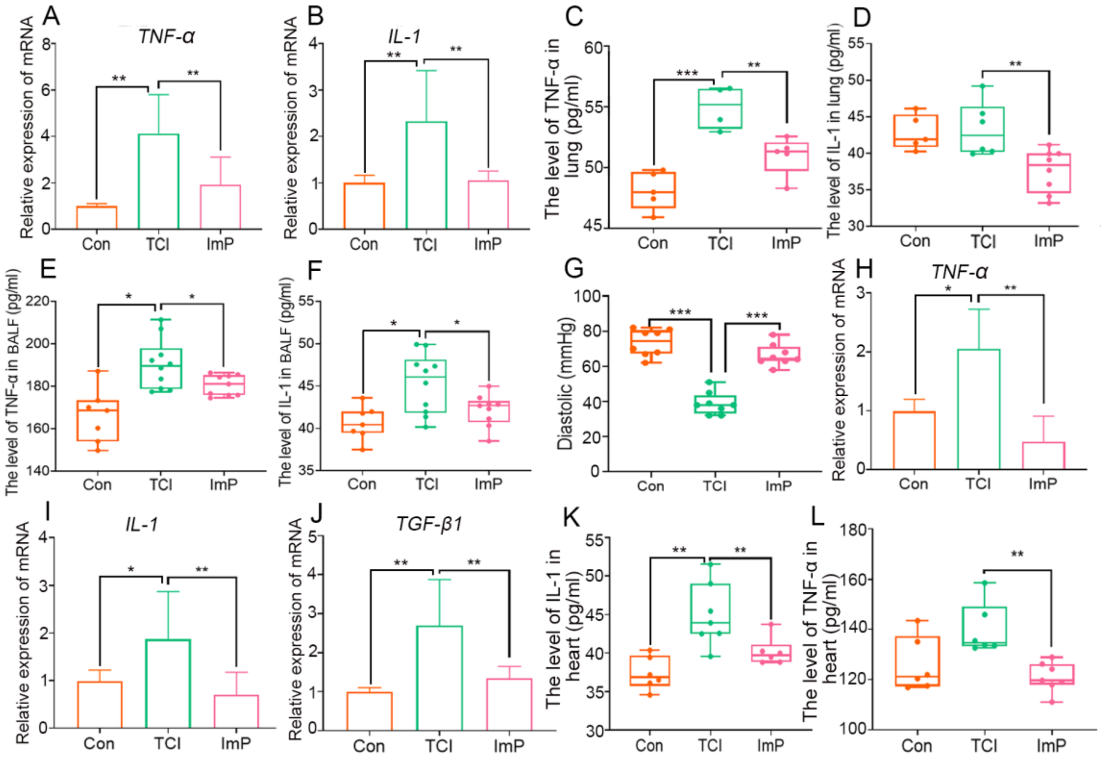

Figure S3. Imidazole propionate ameliorates chest local irradiation-induced toxicity. (A, B) The TNF-α and IL-1 levels in lung tissues of each group mice were assessed by qRT-PCR. (C, D) The TNF-α and IL-1 levels in lung tissues of each group mice were assessed

by ELISA (n=6). (E, F) The TNF-α and IL-1 levels in BALF of each group mice were assessed by ELISA (n=8). (G) The diastolic of each group mice (n=8). (H-J) The TNF-α, IL-1 and TGF-β1 levels in heart tissues of each group mice were assessed by qRT-PCR. (K, L) The IL-1 and TNF-α levels in heart tissues of each group mice were assessed by ELISA (n=6). ($^{*}p < 0.05$, $^{**}p < 0.01$ and $^{***}p<0.001$; Student's *t*-test).

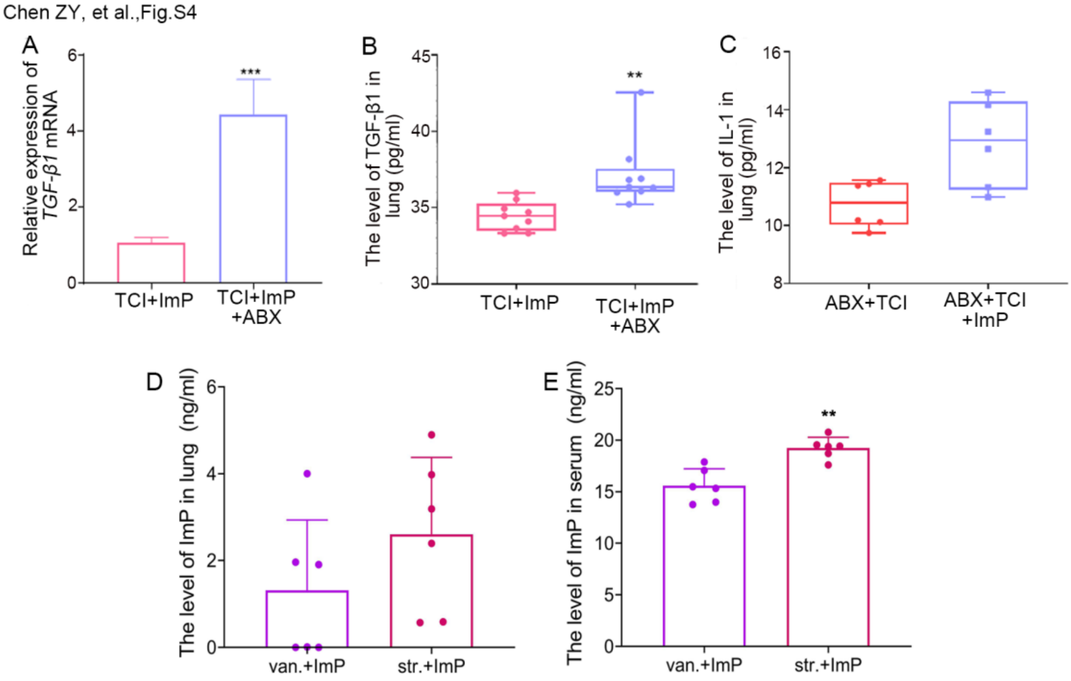

Figure S4. Gut microbiota impacts the assimilation of imidazole propionate. (A) The TGF-β1 levels in lung tissues of each group mice were assessed by qRT-PCR (n=8). (B) The TGF-β1 levels in lung tissues of each group mice were assessed by ELISA (n=8). (C) The IL-1 levels in lung tissues of each group mice were assessed by ELISA (n=6). (D) The ImP levels in lung tissues of vancomycin and streptomycin treat mice group after replenished ImP were assessed by ELISA (n=6). (E) The ImP levels in serum tissues of Vancomycin and Streptomycin treat mice group after replenished ImP were assessed by ELISA (n=6). ($^{*}p < 0.05$, $^{**}p < 0.01$ and $^{***}p<0.001$; Student's *t*-test).

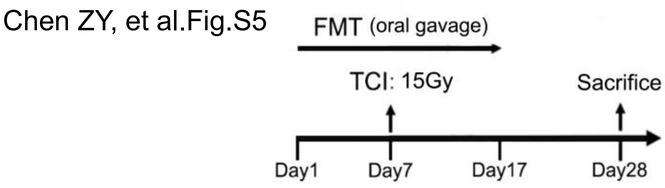

Figure S5. The Schematic diagram of FMT.

Chen ZY, et al.Fig.S6

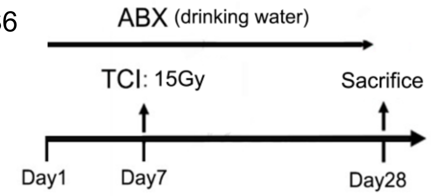

Figure S6. The Schematic diagram of ABX.

4. The whole uncropped images of the original western blots

Western blotting analysis using the following antibodies: NF-κB (1:500, #SC-8008; SANTA CRUZ), GSDMD (1:500, #SC-393581; SANTA CRUZ), caspase-1 (1:500, #SC-56036; SANTA CRUZ), caspase-4 (1:500, #SC-56056; SANTA CRUZ), caspase-5 (1:500, #SC-393346; SANTA CRUZ), GAPDH(1:5000, #60004;Proteintech, USA).

Fig.7F

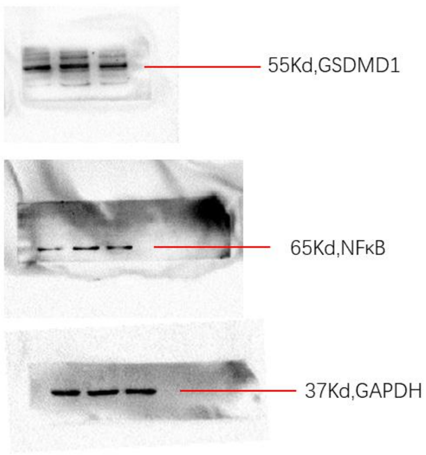

Fig.7G

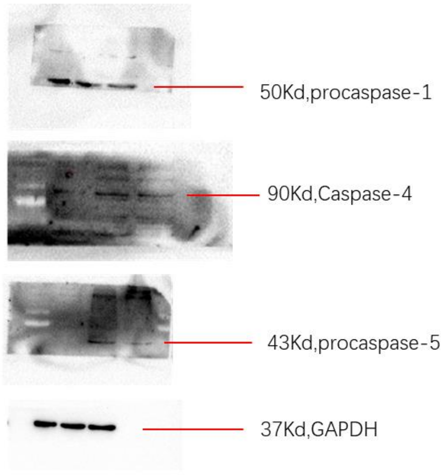

Supplement: Supplementary file 1 [file ijms-22-11436-s001.zip › ijms-1401947-supplementary.pdf]
